# Incidence and risk factors of tocilizumab-induced hypofibrinogenemia in patients with thyroid eye disease: a single-center retrospective study

**DOI:** 10.3389/fendo.2026.1781048

**Published:** 2026-02-20

**Authors:** Ente Wang, Qingyuan Hu, Shanshan Xu, Jianbo Zhou, Jiawei Wang, Zhihui Song, Xinglong Wang

**Affiliations:** 1 Department of Pharmacy, Beijing Tongren Hospital, Capital Medical University, Beijing, China; 2 Department of Pharmacy, Xuanwu Hospital of Capital Medical University, Beijing, China; 3 Department of Endocrinology, Civil Aviation General Hospital, Beijing, China

**Keywords:** adverse drug reaction, hypofibrinogenemia, risk factors, thyroid eye disease, tocilizumab

## Abstract

**Background:**

Tocilizumab, an interleukin-6 receptor antagonist, is increasingly used in moderate-to-severe thyroid eye disease. However, its association with hypofibrinogenemia remains underexplored in this population.

**Methods:**

This single-center retrospective study included 194 TED patients treated with Tocilizumab at Beijing Tongren Hospital between March 2023 and May 2025. Patients were stratified into a positive group (fibrinogen < 1.5 g/L) and a negative group (fibrinogen ≥ 1.5 g/L) based on post-treatment fibrinogen levels. Demographic, clinical, and laboratory data were analyzed to identify risk factors using univariate and multivariate logistic regression.

**Results:**

Among 194 patients, 89 (45.88%) developed hypofibrinogenemia (fibrinogen < 1.5 g/L). The most significant fibrinogen decline occurred after the first Tocilizumab administration (median reduction: 0.88 g/L). Nadir levels were most common before the third (26.4%) or fifth administration (29.6%). Multivariate analysis identified lower baseline fibrinogen (OR = 0.37, P = 0.001), higher body weight (OR = 1.05, P = 0.001), and lower prothrombin time (OR = 0.45, P = 0.008) as independent risk factors.

**Conclusion:**

Hypofibrinogenemia was observed at a higher incidence (45.88%) in thyroid eye disease patients treated with tocilizumab. It typically did not occur immediately after administration but emerged early in the treatment course and persisted with repeated dosing. Baseline fibrinogen level, body weight, and prothrombin time (PT) activity were identified as significant predictors.

## 1 Introduction

Thyroid eye disease (TED), also referred to as thyroid-associated ophthalmopathy (TAO) or Graves’ ophthalmopathy (GO), is an autoimmune inflammatory condition primarily associated with Graves’ disease (1). It is characterized by orbital tissue inflammation, proptosis, and diplopia. In Europe, the estimated prevalence of TED is approximately 100 cases per 100,000 people (2). Corresponding incidence data from the United States in the 2020s show rates of 8.9 and 1.0 cases per 100,000 person-years for women and men, respectively (3). Beyond its epidemiological burden, TED has a substantial impact on patients’ quality of life, frequently causing visual dysfunction and, in moderate−to−severe cases, irreversible vision loss along with considerable psychosocial distress (4).

The autoimmune reaction in GD induces the release of anti-TSH-R autoantibodies (TRAb) by B-cell clones (5), which also recognize TSH receptors expressed on orbital fibroblasts and preadipocytes, triggering an immune−mediated inflammatory cascade. In active GO, a Th1−predominant response leads to infiltration of Th1 lymphocytes (6), which release cytokines like IFN−γ that stimulate orbital fibroblasts to produce Th1 chemokines (CXCL9, CXCL10, CXCL11). These chemokines recruit more inflammatory cells via CXCR3, creating an amplification loop that drives inflammation, tissue expansion. This process results in clinical manifestations such as proptosis, periorbital edema, diplopia, and in severe cases, optic neuropathy.

Conventional management strategies, including supportive measures, corticosteroids, and orbital radiotherapy, often yield suboptimal outcomes with high recurrence rates and significant adverse effects (7). In recent years, biologic agents targeting specific inflammatory pathways have emerged as promising therapeutic options for TED. Tocilizumab (TCZ) is a humanized monoclonal antibody that targets the interleukin−6 receptor (IL−6R). By binding to both soluble and membrane−bound IL−6R, TCZ inhibits IL−6 signaling, thereby blocking downstream pro−inflammatory effects such as immune cell activation, cytokine release, and fibroblast proliferation (8, 9). It has been studied for its ability to modulate the cytokine-mediated inflammation central to TED pathogenesis (10). Several studies (11, 12) have demonstrated its efficacy in reducing disease activity and improving proptosis in patients with moderate-to-severe TED. As evidenced by, randomized controlled trials (13) have reported that tocilizumab significantly reduces clinical activity scores and inflammatory markers compared to placebo, with a favorable short-term safety profile.

Despite its therapeutic potential, safety concerns related to tocilizumab remain. In addition to commonly reported adverse events (such as upper respiratory tract infections, hypercholesterolemia, and elevated liver enzymes), hypofibrinogenemia has emerged as a relatively uncommon but potentially serious adverse reaction. Fibrinogen plays a critical role in coagulation, and reduced levels can increase bleeding risk and potentially lead to life-threatening complications (14). This phenomenon is thought to arise from interleukin-6 inhibition, which disrupts hepatic fibrinogen synthesis, a key component of the acute-phase response (15). Retrospective studies in rheumatology (16) have reported hypofibrinogenemia incidence as high as 46.6% in adults and 76.47% in adolescents with systemic juvenile idiopathic arthritis (17) receiving tocilizumab.

However, although hypofibrinogenemia is well-documented in rheumatologic contexts, its incidence, severity, and risk factors in TED patients remain poorly understood. TED exhibits distinct immunopathological features, often involves comorbid thyroid dysfunction, and focuses on controlling orbital inflammation and fibrosis—factors that may differentiate its safety profile from that of rheumatic diseases. Potential variations may include the incidence of hypofibrinogenemia, dynamics of fibrinogen reduction (e.g., time to onset, extent and duration of decrease, recovery pattern), and influencing factors (e.g., baseline fibrinogen, tocilizumab dosing and treatment duration, concomitant medications and prior therapies). Currently, there is a lack of studies systematically investigating this adverse reaction in the TED population. Therefore, we conducted this study to address this gap.

## 2 Materials and methods

### 2.1 Study design and population

This study is a single-center retrospective observational study that consecutively enrolled patients with thyroid eye disease (TED) treated at Beijing Tongren Hospital, Capital Medical University, between March 2023 and May 2025. The inclusion criteria are as follows: ① meeting the diagnostic criteria for TED in the EUGOGO Consensus; ② having received at least 1 dose of tocilizumab injection (80 mg/4 ml); ③ having at least one record of fibrinogen level monitoring. The exclusion criteria are: ① baseline fibrinogen level < 1.5 g/L before tocilizumab treatment; ② being complicated with liver failure, disseminated intravascular coagulation (DIC), or active hemorrhagic diseases; ③ concomitantly using other biological agents that may significantly affect coagulation function.

Patients were divided into two groups based on the lowest fibrinogen level after treatment: the positive group (fibrinogen < 1.5 g/L) and the negative group (fibrinogen ≥ 1.5 g/L). While the conventional normal range for fibrinogen typically falls between 2.0 and 4.0 g/L, we selected a threshold of <1.5 g/L as the grouping criterion based on established clinical guidelines and consensus statements (18, 19), which recognize this level as a critical threshold associated with a significantly increased risk of bleeding, particularly in perioperative or high-risk settings. This cutoff is clinically relevant for identifying patients who may require monitoring or intervention, even in non-obstetric or non-traumatic contexts, thereby enabling consistent risk stratification and aligning with current evidence-based clinical practices.

### 2.2 Data collection

Data were collected through the Hospital Information System (HIS) and electronic medical records, including demographic and baseline characteristics, laboratory indicators (blood cell count, coagulation parameters, liver & kidney function, metabolic markers and inflammatory marker), and medication history (concomitant medications, pre-tocilizumab treatments, tocilizumab details), as well as the occurrence of bleeding events (The specific items for data collection are listed in **Supplementary Table 1**).

### 2.3 Statistical analysis

Statistical analysis of the data was performed using SPSS 26.0 software. Categorical variables were expressed as frequency and percentage, and group comparisons were conducted using the χ² test or Fisher’s exact test. Continuous variables were first tested for normality. If the data followed a normal distribution (P > 0.05), they were presented as mean ± standard deviation and compared using the independent samples t-test; otherwise, they were expressed as median (Q1, Q3) and compared using the Mann–Whitney U test.

A logistic regression model was employed to investigate risk factors, with the occurrence of ICI-DM as the dependent variable. Variables with P < 0.15 in the univariate analysis were included in the multivariate analysis to identify independent risk factors. A P-value < 0.05 was considered statistically significant.

### 2.4 Ethical considerations

The study protocol was approved by the Ethics Committee of Beijing Tongren Hospital, Capital Medical University. The requirement for informed consent was waived in accordance with the retrospective design, and all patient data were anonymized and handled confidentially.

## 3 Result

### 3.1 Study population and baseline characteristics

A total of 194 patients with thyroid eye disease (TED) treated with tocilizumab were included in this study (**Table 1**). Among them, 70 (36.08%) were male and 124 (63.92%) were female, with a median age of 49 years (interquartile range, IQR: 41.25–57.75). Among all patients, 163 (84.02%) completed 4 dosages of treatment, and 36 (18.56%) completed 6 dosages. Based on the lowest fibrinogen (FIB) level after treatment, 89 patients (45.88%) were divided into the positive group (FIB < 1.5 g/L), and the other 105 patients were divided into the negative group (FIB ≥ 1.5 g/L).

**Table 1:** Basic clinical characteristics and statistically significant differences in patients treated with TCZ.

Variables	Total (n = 194)	Negative group (n = 105)	Positive group (n = 89)	Statistic	*P*
Gender, n(%)				χ²=7.11	0.008
Male	70 (36.08)	29 (27.62)	41 (46.07)		
Female	124 (63.92)	76 (72.38)	48 (53.93)		
Height, M (Q_1_, Q_3_)	164.00 (160.00, 170.00)	162.00 (158.00, 170.00)	165.00 (160.00, 172.25)	Z=-2.52	0.012
Weight (at first administration), M (Q_1_, Q_3_)	65.00 (60.00, 75.00)	63.00 (58.00, 70.00)	70.00 (60.88, 80.00)	Z=-3.14	0.002
Baseline ALT, M (Q_1_, Q_3_)	16.00 (12.00, 23.00)	15.00 (12.00, 22.00)	17.00 (13.00, 24.00)	Z=-1.97	0.049
Baseline PT, M (Q_1_, Q_3_)	11.40 (11.10, 11.80)	11.40 (11.10, 11.90)	11.20 (11.00, 11.60)	Z=-2.34	0.019
Baseline PT Activity, M (Q_1_, Q_3_)	108.10 (101.40, 114.40)	107.80 (99.90, 114.00)	110.00 (102.20, 117.50)	Z=-2.31	0.021
Baseline APTT, M (Q_1_, Q_3_)	26.40 (25.40, 27.78)	26.80 (25.60, 28.10)	26.00 (25.10, 27.30)	Z=-2.42	0.016
Baseline ESR, M (Q_1_, Q_3_)	10.00 (7.00, 16.00)	12.00 (8.00, 18.00)	9.00 (6.00, 13.00)	Z=-3.21	0.001
Baseline Fibrinogen, M (Q_1_, Q_3_)	2.69 (2.37, 3.11)	2.79 (2.48, 3.20)	2.53 (2.24, 2.98)	Z=-2.88	0.004
Initial Dose (MG), n(%)				-	0.016*
300	1 (0.52)	0 (0.00)	1 (1.12)		
320	2 (1.03)	2 (1.90)	0 (0.00)		
360	1 (0.52)	0 (0.00)	1 (1.12)		
400	29 (14.95)	19 (18.10)	10 (11.24)		
440	12 (6.19)	8 (7.62)	4 (4.49)		
460	4 (2.06)	3 (2.86)	1 (1.12)		
480	43 (22.16)	29 (27.62)	14 (15.73)		
500	1 (0.52)	1 (0.95)	0 (0.00)		
520	10 (5.15)	7 (6.67)	3 (3.37)		
540	4 (2.06)	2 (1.90)	2 (2.25)		
560	46 (23.71)	19 (18.10)	27 (30.34)		
580	1 (0.52)	1 (0.95)	0 (0.00)		
600	5 (2.58)	0 (0.00)	5 (5.62)		
620	1 (0.52)	0 (0.00)	1 (1.12)		
640	18 (9.28)	9 (8.57)	9 (10.11)		
680	3 (1.55)	2 (1.90)	1 (1.12)		
720	9 (4.64)	3 (2.86)	6 (6.74)		
780	1 (0.52)	0 (0.00)	1 (1.12)		
800	3 (1.55)	0 (0.00)	3 (3.37)		

Z: Mann-Whitney test, χ²: Chi-square test, -: Fisher exact, *: Simulated p-value.

M: Median, Q_1_: 1st Quartile, Q_3_: 3st Quartile

Significant differences were observed between the two groups in terms of gender (P = 0.008), height (P = 0.012), weight (P = 0.002), baseline mean platelet volume (MPV, P = 0.031), baseline alanine aminotransferase (ALT, P = 0.049), baseline prothrombin time (PT, P = 0.019), baseline PT activity (P = 0.021), baseline Activated Partial Thromboplastin Time (APTT, P = 0.016), baseline erythrocyte sedimentation rate (ESR, P = 0.001), Initial Dose (P = 0.016), and baseline fibrinogen (FIB, P = 0.004) (**Table 1**). No significant differences were observed in other demographic characteristics, comorbidities, or medication history (**Supplementary Table 2**).

### 3.2 Changes in fibrinogen levels

The median baseline FIB level was 2.69 g/L (IQR: 2.37-3.11) for all patients, with 92.8% (180/194) having a baseline FIB≥2 g/L. FIB testing was completed on the day after the first administration in 68 patients, among whom 8 cases (11.76%) had a FIB drop to < 2 g/L (but still > 1.5 g/L). By the time of the second administration, 50 out of these 68 patients (73.53%) had a FIB < 2 g/L, with 15 cases (22.06%) further decreasing to < 1.5 g/L.

Among the 159 patients who completed 4 dosages of treatment and had complete FIB data, the mean lowest FIB value was 1.50 g/L (median: 1.50 g/L, range: 0.90–3.33). The lowest FIB values most frequently occurred before the third administration (n = 42, 26.4%) and before the fifth administration or during follow-up (n = 47, 29.6%) (**Table 2**).

**Table 2:** Timing of the lowest fibrinogen value.

	Before the 2nd admin	Before the 3rd admin	Before the 4th admin	Before the 5th admin/follow up	Before the 6th admin	After the 6th admin
N	25	42	31	47	8	6

**Figure 1:**
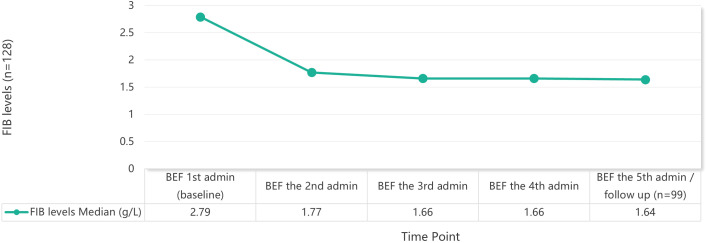
Trend of average FIB levels during TCZ treatment.

After excluding patients who had received exogenous fibrinogen supplementation, the remaining 128 patients who completed 4 treatment dosages showed a continuous decline in median FIB levels, from a baseline of 2.79 g/L to 1.64 g/L before the fifth administration or follow-up. The most pronounced decrease was observed after the first administration, with an median reduction of 0.88 g/L (**Figure 1**).

### 3.3 Risk factors for fibrinogen reduction

Univariate logistic regression analysis indicated that female gender (OR = 0.45, 95% CI: 0.25–0.81, P = 0.008), height (OR = 1.05, 95% CI: 1.01–1.09, P = 0.018), body weight (OR = 1.04, 95% CI: 1.01–1.06, P = 0.003), baseline ALT (OR = 1.03, 95% CI: 1.01–1.06, P = 0.042), baseline prothrombin time (OR = 0.51, 95% CI: 0.30–0.88, P = 0.015), baseline PT activity (OR = 1.03, 95% CI: 1.01–1.06, P = 0.014), baseline activated partial thromboplastin time (OR = 0.84, 95% CI: 0.72–0.97, P = 0.016), baseline erythrocyte sedimentation rate (OR = 0.93, 95% CI: 0.89–0.98, P = 0.003), and baseline fibrinogen (OR = 0.44, 95% CI: 0.26–0.74, P = 0.002) were significantly associated with a reduction in FIB after treatment (**Table 3**).

**Table 3:** Univariate and multivariate logistic analysis results.

Variables	Univariable analysis					Multivariable analysis				
	β	S.E	Z	*P*	OR (95%CI)	β	S.E	Z	*P*	OR (95%CI)
Gender										
Male					1.00 (Reference)					
Female	-0.81	0.30	-2.64	0.008	0.45 (0.25 ~ 0.81)					
Height	0.05	0.02	2.37	0.018	1.05 (1.01 ~ 1.09)					
Weight	0.04	0.01	2.95	0.003	1.04 (1.01 ~ 1.06)	0.05	0.01	3.19	0.001	1.05 (1.02 ~ 1.08)
Baseline ALT	0.03	0.01	2.03	0.042	1.03 (1.01 ~ 1.06)					
Baseline Prothrombin Time	-0.67	0.28	-2.43	0.015	0.51 (0.30 ~ 0.88)	-0.81	0.30	-2.66	0.008	0.45 (0.25 ~ 0.81)
Baseline PT Activity	0.03	0.01	2.45	0.014	1.03 (1.01 ~ 1.06)					
Baseline Activated Partial Thromboplastin Time	-0.18	0.07	-2.41	0.016	0.84 (0.72 ~ 0.97)					
Baseline erythrocyte sedimentation rate	-0.07	0.02	-3.00	0.003	0.93 (0.89 ~ 0.98)					
Baseline Fibrinogen	-0.82	0.27	-3.07	0.002	0.44 (0.26 ~ 0.74)	-1.00	0.31	-3.20	0.001	0.37 (0.20 ~ 0.68)

OR, Odds Ratio; CI, Confidence Interval.

Multivariate analysis further identified the following independent risk factors (**Table 3**): body weight (OR = 1.05, 95% CI: 1.02–1.08, P = 0.001), baseline prothrombin time (OR = 0.45, 95% CI: 0.25–0.81, P = 0.008), and baseline fibrinogen (OR = 0.37, 95% CI: 0.20–0.68, P = 0.001).

## 4 Discussion

This study represents the first comprehensive evaluation of the incidence, temporal dynamics, and risk factors for hypofibrinogenemia induced by tocilizumab (TCZ) in patients with thyroid eye disease (TED). Among 194 TED patients treated with TCZ, 45.88% developed fibrinogen (FIB) levels below 1.5 g/L, a threshold chosen to reflect clinically significant bleeding risk, consistent with He et al.’s (17) approach in systemic juvenile idiopathic arthritis (sJIA). This incidence is notably higher than the 27.1% reported by Cai et al. (20) in other patient cohorts but lower than the 76.47% observed by He et al. in sJIA, highlighting disease-specific differences in the susceptibility to TCZ-induced fibrinogen reduction.

### 4.1 Incidence variability and underlying factors

The variability in hypofibrinogenemia incidence across studies can be attributed to several interconnected factors. First, heterogeneity in inflammatory status and IL-6 activation plays a key role; the localized orbital inflammation in TED contrasts with the systemic cytokine storms characteristic of sJIA or COVID-19, leading to differing baseline IL-6 activity and hepatic acute-phase responses (17, 21). This difference modulates fibrinogen synthesis and the impact of IL-6 blockade. Second, differences in hepatic synthetic function—particularly between adults and children, as seen in sJIA cohorts—may affect fibrinogen production capacity and susceptibility to inhibition (22). Finally, the lack of standardized timing for fibrinogen measurement introduces further variability, as fibrinogen levels fluctuate dynamically following tocilizumab administration.

### 4.2 Dynamic changes in fibrinogen levels

The pattern of FIB decline following TCZ treatment exhibited two key characteristics. Primarily, on the day after the first administration, only 11.76% of patients had FIB drop to <2 g/L (though still >1.5 g/L); however, by the time of before the second administration (approximately 4 weeks later), 73.53% of these patients had FIB <2 g/L, with 22.06% further decreasing to <1.5 g/L. This delayed decline pattern suggests that TCZ likely reduces FIB primarily by inhibiting IL-6 signaling pathway-mediated synthesis rather than by directly promoting its consumption or degradation, consistent with Perl et al.’s (23) findings.

Furthermore, the most pronounced decrease in FIB occurred after the first administration, with an average reduction of 0.90 g/L, representing the largest drop during the entire course; subsequent pre-administration measurements (every 4 weeks) showed average further decreases of 0.06–0.10 g/L. The nadir most frequently occurred before the third administration (26.4%) or before the fifth administration (29.6%). This is consistent with observations in sJIA and RA populations (17, 24), indicating that the inhibitory effect of TCZ on FIB becomes apparent early in treatment and persists with repeated administrations. This temporal pattern suggests that regardless of the disease being treated, TCZ’s suppressive effect on fibrinogen is characterized by an early onset, necessitating intensified monitoring during the initial treatment phase, especially the 2–3 doses.

### 4.3 Mechanistic insights: IL-6 pathway and fibrinogen synthesis

The primary mechanism by which tocilizumab reduces fibrinogen is likely the competitive blockade of IL-6 binding to its receptor. IL-6 is a key cytokine regulating the hepatic acute-phase response, including fibrinogen production. Upon IL-6 binding to its receptor on hepatocytes, the JAK-STAT3 pathway is activated, leading to upregulation of fibrinogen gene transcription (FGA, FGB, FGG) (25, 26). TCZ, a humanized monoclonal antibody targeting the IL-6 receptor, competitively inhibits this interaction, resulting in decreased fibrinogen synthesis (27, 28).

The class effect of IL-6 inhibitors on coagulation is further supported by reports of hypofibrinogenemia associated with other agents such as clazakizumab and olokizumab in clinical trials (29, 30). This reinforces the notion that IL-6 pathway blockade inherently disrupts fibrinogen homeostasis, posing a risk of bleeding complications that clinicians must anticipate.

### 4.4 Risk factors for TCZ-associated hypofibrinogenemia

The multivariate analysis in the current study highlights three independent risk factors for TCZ-associated hypofibrinogenemia (T-HFIB) in TED patients: lower baseline fibrinogen (FIB) levels, higher body weight, and increased prothrombin time (PT) activity. These findings provide important insights into the pathophysiology of T-HFIB and underscore the complexity of risk stratification in different clinical contexts.

The risk factors identified in this TED cohort differ notably from those reported in other populations treated with TCZ. Cai et al. (20) identified infection, COVID-19, CAR-T cell therapy, and concomitant glucocorticoid use as primary risk factors for T-HFIB, reflecting the acute systemic inflammatory and immunosuppressive environment in these patients. For instance, COVID-19 and CAR-T therapies are associated with cytokine release syndrome (CRS), characterized by massive cytokine surges and endothelial dysfunction, which can precipitate coagulation abnormalities including hypofibrinogenemia (20, 31). In contrast, in rheumatoid arthritis (RA) patients, An et al. (24) emphasized disease activity-related markers such as platelet distribution width (PDW), parathyroid hormone (PTH), and bone mineral density (BMD) as critical predictors of T-HFIB. These markers reflect the chronic systemic inflammation and bone metabolism alterations inherent in RA, which influence coagulation homeostasis indirectly. The divergence in risk factors likely arises from fundamental differences in disease pathogenesis. TED is characterized predominantly by orbital fibroblast activation, adipogenesis, and localized inflammation mediated by aberrant autoimmunity against thyroid antigens (21). This local inflammatory milieu contrasts with the systemic immune dysregulation and cytokine storms observed in CRS or RA, where widespread endothelial activation and immune cell perturbations more profoundly affect coagulation pathways (28, 31).

The identification of higher body weight as an independent risk factor in TED patients may be partially explained by the weight-based dosing regimen of TCZ. Heavier patients receive higher absolute doses, potentially increasing the degree of IL-6 receptor blockade and thus the risk of hypofibrinogenemia. This dose-dependent effect aligns with pharmacokinetic data showing increased drug exposure in patients with higher body mass (32). Additionally, obesity itself is a proinflammatory state with altered coagulation profiles, which may exacerbate fibrinogen consumption or impair synthesis (33).

A key commonality between this study and previous reports is the protective role of higher baseline fibrinogen levels against T-HFIB. Cai et al. (20) demonstrated that elevated baseline FIB serves as a buffer against the hypofibrinogenemic effects of TCZ-mediated IL-6 inhibition. This finding is consistent with the biological role of fibrinogen as an acute-phase reactant whose synthesis is IL-6 dependent (34). Patients with higher baseline fibrinogen may possess greater hepatic synthetic reserve or less baseline consumption, enabling better compensation when IL-6 signaling is blocked. This concept is supported by studies in other inflammatory conditions where baseline fibrinogen levels predict the severity of coagulation disturbances (35). It underscores the importance of assessing pre-treatment coagulation parameters to identify patients at risk for hypofibrinogenemia and potentially guide dosing or monitoring strategies.

### 4.5 Bleeding risk of TCZ-associated hypofibrinogenemia

Although a considerable proportion of patients in this study experienced FIB reduction, no obvious bleeding events were observed, which aligns with the conclusion in most literature that “low FIB is mostly asymptomatic” (17, 24, 36). However, it is noteworthy that the risk of bleeding significantly increases in patients with severe hypofibrinogenemia (e.g., <1.0 g/L), particularly in the context of concurrent surgery, trauma, or the use of anticoagulant/antiplatelet drugs (20, 37). Therefore, for TED patients planning to undergo orbital decompression surgery or other invasive procedures, close monitoring of FIB levels during TCZ therapy is recommended.

### 4.6 Clinical implications and limitations of this study

Given the early onset and persistence of TCZ-associated hypofibrinogenemia, particularly in patients with identified risk factors, intensified monitoring of fibrinogen and coagulation parameters during the first 2–3 TCZ doses is warranted. Clinicians should consider baseline fibrinogen and PT testing, adjust dosing in heavier patients if necessary, and maintain vigilance for bleeding events. Moreover, the recognition that hypofibrinogenemia is a class effect of IL-6 inhibitors may guide monitoring protocols across different diseases and biologics, promoting safer use of these agents.

This study has limitations: the single-center retrospective design may introduce bias. Secondly, the dosing intervals and FIB monitoring time points were not entirely uniform, which might affect the accurate capture of the nadir value. Confounding by unmeasured variables, such as nutritional status or subclinical liver dysfunction, cannot be entirely ruled out, despite multivariate adjustments. Future multicenter, prospective studies are recommended to further validate the risk factors identified in this study and to establish a risk prediction model for TCZ-associated hypofibrinogenemia applicable to TED patients. This would provide stronger evidence for clinical safe medication practices.

## 5 Conclusion

Hypofibrinogenemia was observed at a higher incidence (45.88%) in thyroid eye disease patients treated with tocilizumab. It typically did not occur immediately after administration but emerged early in the treatment course and persisted with repeated dosing. Baseline fibrinogen level, body weight, and prothrombin time (PT) activity were identified as significant predictors. Although no bleeding events were observed in this study cohort, severely low fibrinogen levels carry a potential risk of bleeding. Monitoring is therefore recommended, particularly during the initial phase of therapy and before any invasive procedures.

## Data Availability

The raw data supporting the conclusions of this article will be made available by the authors, without undue reservation.
