# Supplementary material for: Incidence and risk factors of tocilizumab-induced hypofibrinogenemia in patients with thyroid eye disease: a single-center retrospective study
Source: Front Endocrinol (Lausanne). 2026 Feb 20;17:1781048. doi: 10.3389/fendo.2026.1781048 (PMC12962926; doi:10.3389/fendo.2026.1781048)
Supplement: Supplementary file 1 [file Table1.docx]

| Category | Items | |
| --- | --- | --- |
| Demographic & Baseline | Gender, age, weight, duration of thyroid dysfunction, duration of TED, smoking history, alcohol consumption history | |
| Laboratory Indicators | Blood cell count | White blood cell count, red blood cell count, platelet count |
|  | Coagulation parameters | Fibrinogen, D-dimer, prothrombin time (PT), activated partial thromboplastin time (APTT), international normalized ratio (INR) (measured before each administration) |
|  | Liver & kidney function | Alanine aminotransferase (ALT), aspartate aminotransferase (AST), creatinine (Cr) |
|  | Metabolic markers | Blood glucose, blood lipids: total cholesterol (TC), triglycerides (TG), low-density lipoprotein cholesterol (LDL-C), high-density lipoprotein cholesterol (HDL-C) |
|  | Inflammatory marker | Erythrocyte sedimentation rate (ESR) |
| Medication History | Concomitant medications | Antithyroid drugs, lipid-lowering drugs, anticoagulant or antiplatelet medications |
|  | Pre-tocilizumab treatments | Use of glucocorticoids or immunosuppressants before tocilizumab treatment |
|  | Tocilizumab details | Cumulative number of administrations and dose of tocilizumab |
| Adverse Events | Occurrence of bleeding events | |

### Suppl.Table 1 Clinical Data Collected for Patients with TED Receiving Tocilizumab
