# Supplementary material for: Incidence and risk factors of tocilizumab-induced hypofibrinogenemia in patients with thyroid eye disease: a single-center retrospective study
Source: Front Endocrinol (Lausanne). 2026 Feb 20;17:1781048. doi: 10.3389/fendo.2026.1781048 (PMC12962926; doi:10.3389/fendo.2026.1781048)
Supplement: Supplementary file 2 [file Table2.docx]

### Suppl. Table 2 Basic clinical characteristics of patients treated with TCZ and Difference Analysis

| Variables | Total (n = 194) | Negative group  (n = 105) | Positive group  1 (n = 89) | Statistic | *P* |
| --- | --- | --- | --- | --- | --- |
|  |  |  |  |  |  |
| Age, M (Q₁, Q₃) | 49.00 (41.25, 57.75) | 53.00 (43.00, 58.00) | 46.00 (40.00, 56.00) | Z=-1.42 | 0.154 |
| Height, M (Q₁, Q₃) | 164.00 (160.00, 170.00) | 162.00 (158.00, 170.00) | 165.00 (160.00, 172.25) | Z=-2.52 | **0.012** |
| Weight (at first administration), M (Q₁, Q₃) | 65.00 (60.00, 75.00) | 63.00 (58.00, 70.00) | 70.00 (60.88, 80.00) | Z=-3.14 | **0.002** |
| Course of abnormal thyroid function at first medication (months), M (Q₁, Q₃) | 18.00 (11.25, 36.00) | 20.00 (11.50, 42.00) | 15.00 (11.00, 36.00) | Z=-0.72 | 0.474 |
| Course of thyroid eye disease at first medication (months), M (Q₁, Q₃) | 8.00 (4.00, 12.00) | 7.00 (4.00, 12.00) | 8.00 (5.00, 12.00) | Z=-0.41 | 0.682 |
| Baseline White blood cell level, M (Q₁, Q₃) | 6.00 (5.00, 7.20) | 6.00 (5.00, 6.80) | 6.03 (5.04, 7.52) | Z=-0.87 | 0.385 |
| Baseline Absolute neutrophil count, M (Q₁, Q₃) | 3.36 (2.71, 4.39) | 3.37 (2.65, 4.26) | 3.35 (2.83, 4.66) | Z=-0.66 | 0.507 |
| Baseline Platelets, M (Q₁, Q₃) | 239.00 (205.00, 275.00) | 240.50 (208.75, 282.25) | 238.00 (200.00, 267.00) | Z=-1.49 | 0.137 |
| Baseline Mean platelet volume, M (Q₁, Q₃) | 10.00 (9.40, 10.62) | 9.90 (9.38, 10.40) | 10.10 (9.50, 10.80) | Z=-1.84 | 0.066 |
| Baseline Fasting blood glucose, M (Q₁, Q₃) | 4.85 (4.40, 5.30) | 4.80 (4.40, 5.20) | 4.90 (4.50, 5.40) | Z=-0.96 | 0.336 |
| Baseline Creatinine, M (Q₁, Q₃) | 63.00 (55.25, 73.00) | 62.00 (54.00, 73.00) | 63.00 (58.00, 73.00) | Z=-0.90 | 0.365 |
| Baseline ALT (Alanine Aminotransferase), M (Q₁, Q₃) | 16.00 (12.00, 23.00) | 15.00 (12.00, 22.00) | 17.00 (13.00, 24.00) | Z=-1.97 | **0.049** |
| Baseline AST (Aspartate Aminotransferase), M (Q₁, Q₃) | 18.00 (15.00, 21.00) | 18.00 (15.00, 21.00) | 18.00 (15.00, 21.00) | Z=-0.45 | 0.652 |
| Baseline TC (Total Cholesterol), M (Q₁, Q₃) | 4.53 (4.00, 5.04) | 4.69 (4.07, 5.20) | 4.45 (3.96, 4.99) | Z=-1.64 | 0.101 |
| Baseline TG (Triglycerides), M (Q₁, Q₃) | 1.20 (0.90, 1.61) | 1.17 (0.91, 1.59) | 1.23 (0.90, 1.61) | Z=-0.87 | 0.384 |
| Baseline LDL-c (Low-Density Lipoprotein Cholesterol), M (Q₁, Q₃) | 2.70 (2.17, 3.22) | 2.76 (2.26, 3.21) | 2.54 (2.05, 3.22) | Z=-1.08 | 0.280 |
| Baseline HDL-c (High-Density Lipoprotein Cholesterol), M (Q₁, Q₃) | 1.23 (1.04, 1.49) | 1.25 (1.06, 1.63) | 1.22 (1.01, 1.42) | Z=-1.80 | 0.072 |
| Baseline Creatine Kinase level, M (Q₁, Q₃) | 65.50 (47.25, 93.00) | 67.00 (51.00, 91.00) | 61.00 (41.00, 96.00) | Z=-0.78 | 0.434 |
| Baseline Prothrombin Time, M (Q₁, Q₃) | 11.40 (11.10, 11.80) | 11.40 (11.10, 11.90) | 11.20 (11.00, 11.60) | Z=-2.34 | **0.019** |
| Baseline PT Activity, M (Q₁, Q₃) | 108.10 (101.40, 114.40) | 107.80 (99.90, 114.00) | 110.00 (102.20, 117.50) | Z=-2.31 | **0.021** |
| Baseline International Normalized Ratio, M (Q₁, Q₃) | 0.96 (0.93, 1.00) | 0.96 (0.93, 1.00) | 0.95 (0.93, 0.99) | Z=-1.42 | 0.155 |
| Baseline Activated Partial Thromboplastin Time, M (Q₁, Q₃) | 26.40 (25.40, 27.78) | 26.80 (25.60, 28.10) | 26.00 (25.10, 27.30) | Z=-2.42 | **0.016** |
| Baseline D-Dimer, M (Q₁, Q₃) | 0.17 (0.11, 0.26) | 0.16 (0.12, 0.26) | 0.17 (0.10, 0.27) | Z=-0.29 | 0.775 |
| Baseline Fibrinogen Degradation Products, M (Q₁, Q₃) | 2.50 (2.50, 2.50) | 2.50 (2.50, 2.50) | 2.50 (2.50, 2.50) | Z=-0.98 | 0.325 |
| Baseline Antithrombin III, M (Q₁, Q₃) | 90.90 (84.05, 100.28) | 91.80 (84.03, 101.40) | 89.45 (84.15, 98.70) | Z=-1.24 | 0.215 |
| Baseline Interleukin-6, M (Q₁, Q₃) | 3.45 (2.05, 4.49) | 3.46 (1.89, 4.46) | 3.35 (2.34, 4.49) | Z=-0.39 | 0.697 |
| Baseline Interleukin-5, M (Q₁, Q₃) | 2.71 (2.11, 3.10) | 2.75 (1.96, 3.03) | 2.70 (2.19, 3.30) | Z=-0.94 | 0.346 |
| Baseline Erythrocyte Sedimentation Rate, M (Q₁, Q₃) | 10.00 (7.00, 16.00) | 12.00 (8.00, 18.00) | 9.00 (6.00, 13.00) | Z=-3.21 | **0.001** |
| Baseline Fibrinogen, M (Q₁, Q₃) | 2.69 (2.37, 3.11) | 2.79 (2.48, 3.20) | 2.53 (2.24, 2.98) | Z=-2.88 | **0.004** |
| Gender, n(%) |  |  |  | χ²=7.11 | **0.008** |
| Male | 70 (36.08) | 29 (27.62) | 41 (46.07) |  |  |
| Female | 124 (63.92) | 76 (72.38) | 48 (53.93) |  |  |
| Thyroid function status at first medication, n(%) |  |  |  | χ²=1.17 | 0.558 |
| Hypothyroidism | 36 (18.56) | 20 (19.05) | 16 (17.98) |  |  |
| Hyperthyroidism | 148 (76.29) | 78 (74.29) | 70 (78.65) |  |  |
| Euthyroid | 10 (5.15) | 7 (6.67) | 3 (3.37) |  |  |
| Hypertension, n(%) |  |  |  | χ²=0.78 | 0.376 |
| No | 139 (71.65) | 78 (74.29) | 61 (68.54) |  |  |
| Yes | 55 (28.35) | 27 (25.71) | 28 (31.46) |  |  |
| Diabetes Mellitus, n(%) |  |  |  | χ²=0.23 | 0.631 |
| No | 132 (68.04) | 73 (69.52) | 59 (66.29) |  |  |
| Yes | 62 (31.96) | 32 (30.48) | 30 (33.71) |  |  |
| Dyslipidemia, n(%) |  |  |  | χ²=1.40 | 0.236 |
| No | 107 (55.15) | 62 (59.05) | 45 (50.56) |  |  |
| Yes | 87 (44.85) | 43 (40.95) | 44 (49.44) |  |  |
| Rheumatoid Arthritis, n(%) |  |  |  | - | 0.459 |
| No | 193 (99.48) | 105 (100.00) | 88 (98.88) |  |  |
| Yes | 1 (0.52) | 0 (0.00) | 1 (1.12) |  |  |
| Smoking Status, n(%) |  |  |  | χ²=1.82 | 0.178 |
| No | 142 (73.20) | 81 (77.14) | 61 (68.54) |  |  |
| Yes | 52 (26.80) | 24 (22.86) | 28 (31.46) |  |  |
| Drinking Status, n(%) |  |  |  | χ²=0.22 | 0.636 |
| No | 166 (85.57) | 91 (86.67) | 75 (84.27) |  |  |
| Yes | 28 (14.43) | 14 (13.33) | 14 (15.73) |  |  |
| Concomitant Lipid-Lowering Drugs, n(%) |  |  |  | χ²=0.04 | 0.845 |
| No | 130 (67.01) | 71 (67.62) | 59 (66.29) |  |  |
| Yes | 64 (32.99) | 34 (32.38) | 30 (33.71) |  |  |
| Concomitant Antiplatelet/Anticoagulant Drugs, n(%) |  |  |  | χ²=0.12 | 0.730 |
| No | 178 (91.75) | 97 (92.38) | 81 (91.01) |  |  |
| Yes | 16 (8.25) | 8 (7.62) | 8 (8.99) |  |  |
| Concomitant Antithyroid Drugs, n(%) |  |  |  | χ²=0.86 | 0.354 |
| No | 61 (31.44) | 36 (34.29) | 25 (28.09) |  |  |
| Yes | 133 (68.56) | 69 (65.71) | 64 (71.91) |  |  |
| Concomitant Antihypothyroid Drugs, n(%) |  |  |  | χ²=0.04 | 0.848 |
| No | 158 (81.44) | 85 (80.95) | 73 (82.02) |  |  |
| Yes | 36 (18.56) | 20 (19.05) | 16 (17.98) |  |  |
| Previous Immunosuppressant Use Before Administration, n(%) |  |  |  | χ²=1.85 | 0.174 |
| No | 140 (72.16) | 80 (76.19) | 60 (67.42) |  |  |
| Yes | 54 (27.84) | 25 (23.81) | 29 (32.58) |  |  |
| Previous Systemic Hormone Use Before Administration, n(%) |  |  |  | χ²=1.40 | 0.236 |
| No | 107 (55.15) | 62 (59.05) | 45 (50.56) |  |  |
| Yes | 87 (44.85) | 43 (40.95) | 44 (49.44) |  |  |
| Treatment Course, n(%) |  |  |  | χ²=7.75 | 0.171 |
| 1 | 16 (8.25) | 12 (11.43) | 4 (4.49) |  |  |
| 2 | 7 (3.61) | 2 (1.90) | 5 (5.62) |  |  |
| 3 | 11 (5.67) | 8 (7.62) | 3 (3.37) |  |  |
| 4 | 108 (55.67) | 54 (51.43) | 54 (60.67) |  |  |
| 5 | 17 (8.76) | 8 (7.62) | 9 (10.11) |  |  |
| 6 | 35 (18.04) | 21 (20.00) | 14 (15.73) |  |  |
| Initial Dose (MG), n(%) |  |  |  | - | **0.016*** |
| 300 | 1 (0.52) | 0 (0.00) | 1 (1.12) |  |  |
| 320 | 2 (1.03) | 2 (1.90) | 0 (0.00) |  |  |
| 360 | 1 (0.52) | 0 (0.00) | 1 (1.12) |  |  |
| 400 | 29 (14.95) | 19 (18.10) | 10 (11.24) |  |  |
| 440 | 12 (6.19) | 8 (7.62) | 4 (4.49) |  |  |
| 460 | 4 (2.06) | 3 (2.86) | 1 (1.12) |  |  |
| 480 | 43 (22.16) | 29 (27.62) | 14 (15.73) |  |  |
| 500 | 1 (0.52) | 1 (0.95) | 0 (0.00) |  |  |
| 520 | 10 (5.15) | 7 (6.67) | 3 (3.37) |  |  |
| 540 | 4 (2.06) | 2 (1.90) | 2 (2.25) |  |  |
| 560 | 46 (23.71) | 19 (18.10) | 27 (30.34) |  |  |
| 580 | 1 (0.52) | 1 (0.95) | 0 (0.00) |  |  |
| 600 | 5 (2.58) | 0 (0.00) | 5 (5.62) |  |  |
| 620 | 1 (0.52) | 0 (0.00) | 1 (1.12) |  |  |
| 640 | 18 (9.28) | 9 (8.57) | 9 (10.11) |  |  |
| 680 | 3 (1.55) | 2 (1.90) | 1 (1.12) |  |  |
| 720 | 9 (4.64) | 3 (2.86) | 6 (6.74) |  |  |
| 780 | 1 (0.52) | 0 (0.00) | 1 (1.12) |  |  |
| 800 | 3 (1.55) | 0 (0.00) | 3 (3.37) |  |  |
| Z: Mann-Whitney test, χ²: Chi-square test, -: Fisher exact, *: Simulated p-value | | | | | |
| M: Median, Q₁: 1st Quartile, Q₃: 3st Quartile | | | | | |
